# Enhanced expression of thioredoxin‐interacting‐protein regulates oxidative DNA damage and aging

**DOI:** 10.1002/1873-3468.13156

**Published:** 2018-06-27

**Authors:** Tina Oberacker, Jörg Bajorat, Sabine Ziola, Anne Schroeder, Daniel Röth, Lena Kastl, Bruce A. Edgar, Wolfgang Wagner, Karsten Gülow, Peter H. Krammer

**Affiliations:** ^1^ Tumor Immunology Program (D030) German Cancer Research Center (DKFZ) Heidelberg Germany; ^2^ German Cancer Research Center (DKFZ) Center for Molecular Biology University of Heidelberg Alliance Germany; ^3^ Huntsman Cancer Institute University of Utah Salt Lake City UT USA; ^4^ Department for Stem Cell Biology and Cellular Engineering Helmholtz‐Institute for Biomedical Engineering RWTH Aachen University Medical School Germany; ^5^ Internal Medicine I University Hospital Regensburg Germany

**Keywords:** aging, DNA damage, oxidative stress, reactive oxygen species, thioredoxin

## Abstract

The “free radical theory of aging” suggests that reactive oxygen species (ROS) are responsible for age‐related loss of cellular functions and, therefore, represent the main cause of aging. Redox regulation by thioredoxin‐1 (TRX) plays a crucial role in responses to oxidative stress. We show that thioredoxin‐interacting protein (TXNIP), a negative regulator of TRX, plays a major role in maintaining the redox status and, thereby, influences aging processes. This role of TXNIP is conserved from flies to humans. Age‐dependent upregulation of TXNIP results in decreased stress resistance to oxidative challenge in primary human cells and in *Drosophila*. Experimental overexpression of TXNIP in flies shortens lifespan due to elevated oxidative DNA damage, whereas downregulation of TXNIP enhances oxidative stress resistance and extends lifespan.

## Abbreviations

**Dox**, Doxycycline

**EV**, empty vector

**FACS**, fluorescence‐activated cell scanning

**H**_**2**_ **O**_**2**_, hydrogen peroxide

**HPC**, hematopoietic progenitor cells

**MFI**, mean fluorescence intensity

**NAC**, N‐Acyetyl‐Cystein

**PI**, propidium‐iodide

**PQ**, paraquat

**ROS**, reactive oxygen species

**RT**, room temperature

**TRX**, Thioredoxin

**TXNIP**, Thioredoxin‐Interacting‐Protein

**VDUP1**, Vitamin‐D_3_‐Upregulated‐Protein 1

Aging is a universal progressive process that occurs to different degrees in all individuals and species. Since the population of the developed world is aging with major implications for health care resources and the productive workforce, it is of general interest to gain a better understanding of the molecular mechanisms of aging and to develop strategies to improve healthy lifespan. Harman hypothesized in “the free radical theory of aging” that the major factor contributing to aging and organ malfunction is accumulation of oxidative damage of macromolecules (e.g. lipids, proteins and DNA) over time 1, 2, 3, 4. One well accepted determinator of aging is accumulation of genetic damage throughout life 5. Moreover, various premature aging diseases are the consequence of accumulation of DNA damage 6. The integrity and stability of DNA are continuously challenged by ROS 7. Interestingly, age‐dependent dysregulation of the redox system and accumulation of DNA damage is connected by the cellular anti‐oxidant TRX. It has been shown that TRX is involved in DNA replication and DNA repair 8, 9. Further studies demonstrated that redox regulation by TRX protected against aging and age‐related diseases 10. Overexpression of TRX in transgenic mice or in transgenic *Drosophila melanogaster* leads to lifespan extension 11, 12, 13. Otherwise, loss of TRX leads to a decrease in lifespan as shown in *C. elegans* as well as in flies 14, 15. Down regulation of TRX in mice showed no beneficial effect on lifespan. However, these studies demonstrate that reduced levels of TRX may be more important for tumor development than aging 10. Since, TRX levels remain constant during life we speculated that the activity of TRX is regulated by its natural inhibitor TXNIP during aging. TXNIP, also known as Vitamin‐D_3_‐Upregulated‐Protein 1 (VDUP1), is a member of the α‐arrestin family 16. Here, we show for the first time that the TRX inhibitor TXNIP is upregulated during aging in primary human cells and *Drosophila melanogaster*. Thus, we elucidate a novel mechanism conserved from fly to man showing that age‐dependent upregulation of TXNIP induces a perturbation of the intracellular redox equilibrium. TXNIP upregulation leads to accumulation of ROS and, concordantly, to an increase in oxidative DNA damage, both crucial hallmarks of aging. We demonstrate that in *Drosophila* increased TXNIP expression leads to induction of DNA damage and, therefore, to a significant reduction in median lifespan, whereas decreased TXNIP expression results in prolonged median lifespan due to lower oxidative DNA damage.

## Materials and methods

### Chemicals

Chemicals were obtained from Sigma‐Aldrich unless otherwise indicated. Hygromycin B was obtained from GERBU.

### Cell culture conditions

Jurkat J16‐145 is a subclone of Jurkat J16 17. Jurkat T cells were cultured in RPMI 1640 containing 10% FCS. Primary human T cells were cultured at a concentration of 2 × 10^6^ cells·mL^−1^ in RPMI 1640 supplemented with 10% FCS.

*Drosophila* Schneider‐2 cells (S2) were cultured in Schneider's insect medium (Sigma‐Aldrich, Darmstadt, Germany) supplemented with 10% (v/v) FCS at room temperature (RT). Clones were selected using hygromycin B (400 μg·mL^−1^).

### Blood donors

T cells were isolated from the blood of healthy human donors at the age of 20–25 years (*n* = 7) and above 55 years old (*n* = 16). Informed consent was obtained from all subjects before inclusion in the study. The study was conducted according to the ethical guidelines of the German Cancer Research Center and the Helsinki Declaration, and it was approved by the ethics committee II of the Ruprecht‐Karls‐University of Heidelberg, Germany.

### Isolation of human peripheral T cells

Primary human T cells were purified as described 17. Purity of the prepared T cells was verified by staining with PE‐conjugated anti‐CD3 antibodies followed by fluorescence‐activated cell scanning (FACS) analysis.

### Gene expression analysis in human hematopoietic progenitor cells

CD34^+^ cells were isolated from cord blood or mobilized peripheral blood of 15 healthy donors between 27 and 73 years and analyzed by Affymetrix technology as described 18.

### Generation of stable TXNIP knockdown

For production of lentiviral particles, HEK293T cells, pretreated with 25 μm chloroquine for 1 h, were transfected with vectors containing the shRNA against TXNIP (Open Biosystems, Heidelberg) and a plasmid mixture for *gag*,* pol*,* env* and VSV‐G for pseudotyping. 8 h post transfection medium was replaced from packaging cells. After 2 days, the supernatant was passed through a 0.45 μm filter, supplemented with Polybrene (8 μg·mL^−1^). 1x10^5^ target cells were infected by spin occulation with 1 mL of viral supernatant. Stably transduced Jurkat cells were selected by puromycin (1 μg·mL^−1^) and Doxycycline (Dox)‐dependent shRNA expression was checked by Western blot analysis.

### Generation of a Drosophila α‐TXNIP monoclonal antibody

A partial sequence (AS 2‐177) derived from TXNIP cDNA (RE 65531, DGRC) was used for immunization of BALB/c mice. B cells from reactive mice were isolated and fused to myeloma cells to obtain hybridoma cells. Antibody‐secretion of hybridoma cells was tested by ELISA and Western blot analysis. Positive cells were subcloned two times. Anti‐monoclonal *Drosophila* TXNIP antibody was prepared from hybridoma supernatants by Protein A affinity purification.

### Transfection of S2‐cells

Transfection of S2 cells was performed using Ca_3_(PO_4_)_2_ according to manufacturer's instructions (Life Technologies, Darmstadt, Germany). To ensure stable overexpression, in addition to the *TXNIP*‐pAc 5.1/V5‐HisA, cotransfection with pCo Hygro (400 μg·mL^−1^) was conducted.

### Generation of S2 overexpressing TXNIP

To generate S2 cells overexpressing TXNIP (OE‐TXNIP), *TXNIP* was amplified by PCR from the cDNA clone (RE 65531, DGRC). The 5′‐primer was modified to introduce an *EcoRV* restriction site, whereas the 3′‐primer was modified to reconstitute a stop codon and to introduce an *XhoI* restriction site. The PCR product was cloned into pAc 5.1/V5‐HisA (Life Technologies). The corresponding plasmid pAc 5.1/V5‐HisA without cDNA served as empty vector control (EV).

### Quantitative PCR (qPCR)

Total RNA was isolated from cells, with the RNeasy Mini Kit (Qiagen, Hilden, Germany). RNA from fly heads was isolated with TRIzol reagent (Life Technologies) according to the manufacturer's instructions. RNA was reverse transcribed and qPCR performed as described previously 19. The housekeeping gene GAPDH (human samples) and *rp49* (*Drosophila* samples) was used as control gene for normalization. For primer sequences Table S1.

### Determination of ROS generation

Cells were stained with 5 μm H_2_DCF‐DA (Life Technologies) for 10 min for human samples and 30 min for S2 cells. Cells were washed twice with ice‐cold PBS and ROS generation was determined by FACS analysis. ROS generation was quantified as the increase in mean fluorescence intensity (MFI) calculated as reported previously 20.

### Thioredoxin (TRX) activity assay

Analysis of TRX activity was assessed in protein lysates of primary T cells as described previously 21, 22. The fluorescence‐based activity assay FkTRX‐02 (BIOZOL Diagnostica) was performed according to manufacturer's instructions. For S2 as well as for whole fly lysates the Thioredoxin Activity Fluorescent Assay Kit (Cayman) was performed according to manufacturer's instructions. Here the method is based on the reduction of insulin by reduced TRX.

### Assessment of cell death

Cell death of human cells was assessed as the decrease in the forward‐to‐side scatter profile compared to living cells and recalculated to “specific cell death,” as described previously 17.

Cell death of S2 was analyzed by flow cytometry (FACSCanto II, Becton Dickinson) by determining percentage of propidium‐iodide (PI) positive cells (ex488/em617). 5 × 10^5^ cells were collected and once washed with PBS. After washing, cells were stained with PI (2 μg·mL^−1^) and incubated for 15 min at RT.

### Immunofluorescence of S2 cells

Immunofluorescence was performed as described 23. In brief, 10^5^ cells·mL^−1^ were washed once with PBS and plated in 24‐well plates at RT overnight. The next day, the cells were left untreated, treated with H_2_O_2_ (20 mm), NAC (20 mm) or Trolox (50 μm) for 2 h. Next, cells were transferred into a microfuge tube and centrifuged at 1.550 r.p.m. for 5 min, washed with PBS and resuspended in 1 mL PBS. Anti‐ y2HAv (UNC93‐5.2.1, 1 : 1000, Developmental Studies Hybridoma Bank, DSHB, University of Iowa) was used as primary antibody. For detection a mouse anti‐ALF488 (1 : 200, Molecular probes) and Hoechst33342 (1 : 500, Life Technologies) were used. Immunofluorescent stainings were analyzed by confocal microscopy (LSM710, Zeiss).

### SDS/PAGE and Western blot

For Western blot analysis, whole cell extracts were prepared from cells by lysing in ice‐cold RIPA lysis buffer (50 mm Tris‐HCl, pH 8.0, 120 mm NaCl, 1% NP‐40, 0.5% Na‐Desoxycholat, 0.1% SDS, 2 mm EDTA, 25 mm NaF, 0.2 mm NaVO_4_, 1 mm DTT, and complete protease inhibitor cocktail from Roche) for 30 min on ice. Fly extracts were generated as described 24. The following antibodies were used: anti‐human TXNIP (1 : 10 000), anti‐actin (1 : 8000, Acris) or anti‐*Drosophila* TXNIP (1 : 500, generated for this study), anti‐β‐tubulin (E7, 1 : 1000, DSHB), anti‐V5 (1 : 5000, Life Technologies) and anti‐y‐H2AV (UNC93‐5.2.1, 1 : 500, DSHB). For quantitative analysis of TXNIP expression signals were quantified with ImageJ 1.48v.

### Fly strains and husbandry

The following strains of *Drosophila melanogaster* were used: isogenic *w* ^*1118*^ (here referred to as wild type WT, Bloomington Stock Center), *yw*; +;P[UAS‐*TXNIP*] 25, *w* ^*1118;*^ P[UAS‐*TXNIP*‐RNAi]; + (VDRC, 15203) 26, *w* ^*1118;*^+; P[*tub‐GAL4*]/*TM6B* and *w* ^*1118;*^ *CyO/IF; MRKS (Sb)/TM6B*. To generate *w* ^*1118*^; +; P[UAS‐*TXNIP*] stock *yw*; +; P[UAS‐*TXNIP*] flies were crossed to *w* ^*1118*^ *; CyO/IF; MRKS (Sb)/TM6B* flies. Thereafter, *w* ^*1118*^ *; +;* P[UAS‐*TXNIP*] and *w* ^*1118*^ *;* P[UAS‐*TXNIP*‐RNAi]; + was backcrossed *w* ^*1118*^ using standard *Drosophila* genetics. For downregulation or overexpression of *TXNIP* males of the strain *w* ^*1118*^; +; P[*tub‐GAL4*]/TM6B were crossed to *w* ^*1118*^ *; +;* P[UAS‐*TXNIP*] or *w* ^*1118*^; P[UAS‐*TXNIP*‐RNAi]; + females, respectively. Flies were raised at 25 °C (constant humidity and 12 h light‐dark cycle) on standard corn meal food. If not otherwise stated, flies were used for analysis 4 days after eclosion.

### Lifespan studies

Age‐matched flies were collected as previously described 27. In brief, *w* ^*1118*^; P[UAS‐*TXNIP*‐RNAi]; + or *w* ^*1118*^ *; +;* P[UAS‐*TXNIP*] and *w* ^*1118*^ *;* +; + females were crossed to *w* ^*1118*^; +; P[*tub‐GAL4*]/*TM6B* males. Flies of the F1 generation were mated for 3 days and, thereafter, females were collected and used for analysis. Flies were transferred to fresh vials and dead flies were counted every 2nd or 3rd day. The three genotypes were scored in parallel.

### Paraquat treatment (analysis of resistance towards oxidative stress)

200 female flies per genotype were used to determine oxidative stress resistance. Flies were transferred to empty vials and starved for 3 h before paraquat (PQ, a redox‐cycling agent) treatment was started. Paraquat (150 μL, 15 mm in 5% sucrose or 5% sucrose for control flies) was supplied on filter paper. Dead flies were counted every 6 to 12 h. The three genotypes were scored in parallel.

### Starvation stress treatment

For starvation stress determinations 150 female flies per genotype were used. Flies were transferred into vials containing a filter paper soaked with 150 μL of water. Dead flies were counted every 6–12 h. The three genotypes were scored in parallel.

### Statistics

Unpaired student's *t*‐tests were used to compare samples derived from young and aged donors as well as empty vector (EV) control transfected and OE‐TXNIP S2 cells. Welch's *t*‐tests were used to calculate statistics for TRX activity and Log‐Rank test for lifespan analysis. *P* values were calculated using GraphPad Prism 4 or the Sigma Plot 13 software package.

## Results

### TXNIP is upregulated in various human tissues during aging

**Figure 1:**
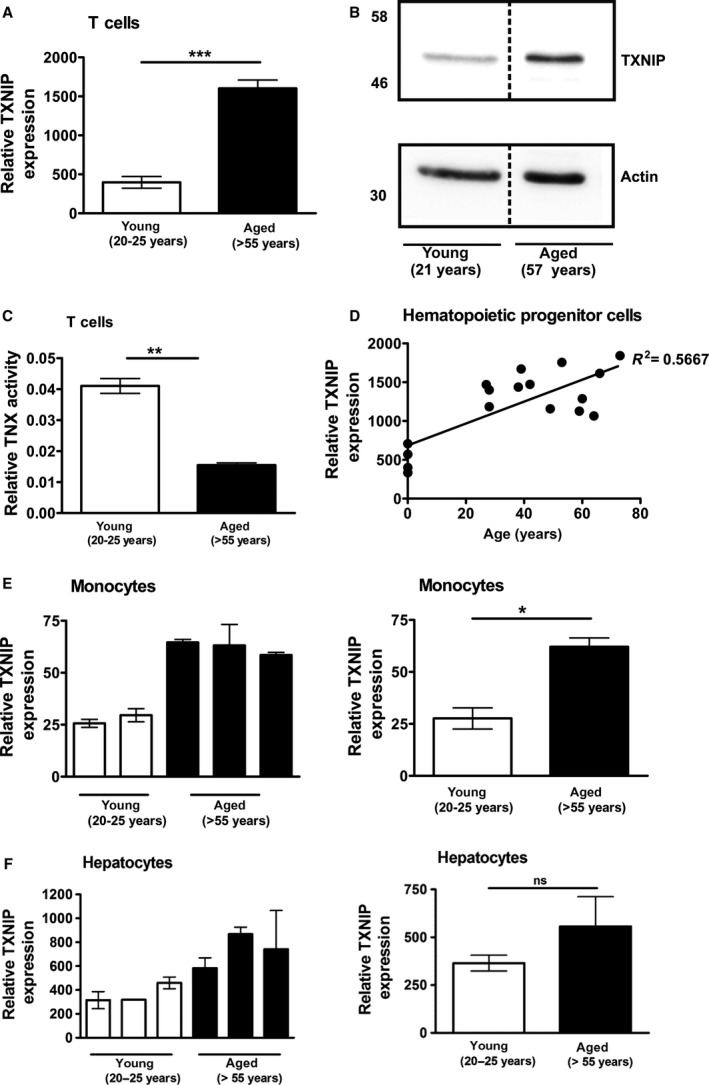
Enhanced TXNIP expression in different human cell types. (A‐F) Cells were isolated from young and aged donors. (A) T cells from aged donors show enhanced TXNIP expression compared to young donors as determined by qPCR. Statistical differences were determined by an unpaired student's *t*‐test (mean ± SEM, *n* = 7 young donors, *n* = 16 aged donors, ***P < 0.001). (B) Representative Western blot of T cells from a young (21 years) and aged donor (57 years). The diagram shows the quantification of the representative Western blot. TXNIP signal was divided by the value of the respective actin signal and normalized to the young donor. (C) Thioredoxin (TRX) activity is decreased in T cells of aged individuals. Statistical differences were determined by an unpaired student's *t*‐test (mean ± SEM, *n* = 3, **P < 0.01). (D) Affymetrix gene chip array analysis revealed higher TXNIP expression in hematopoietic progenitor cells (HPC) isolated from aged donors compared to young donors. (E,F) TXNIP mRNA expression of monocytes (E) and hepatocytes (F) from young and aged donors are shown as bar diagrams representing single individuals (left panels, measured as triplicates) or as mean values (right panels). Statistical differences were determined by an unpaired student's *t*‐test (mean ± SEM, *n* = 3 except for young donors monocytes (*n* = 2), not significant (ns): *P* > 0.05; *P < 0.05).

**Figure 2:**
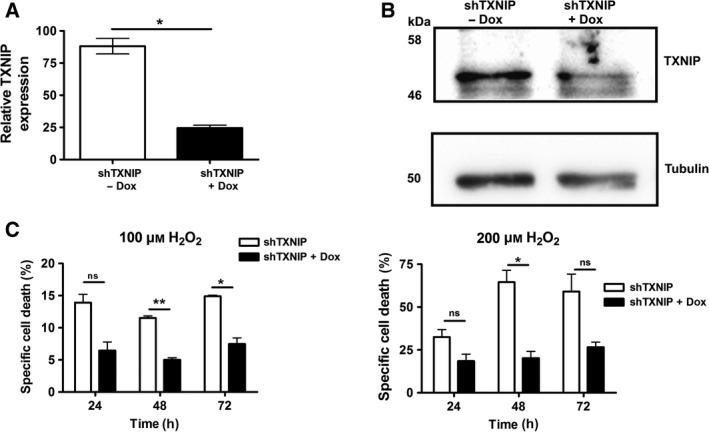
TXNIP knockdown leads to increased oxidative stress resistance. (A‐C) Jurkat T cells were stably transduced with an inducible shRNA construct against TXNIP (shTXNIP). Cells were either left untreated or treated with 1 μg**·**mL^−1^ Doxycycline (Dox) for 24 h and TXNIP expression was analyzed by qPCR (A) or Western blot analysis (B). (C) Cell death was induced by treatment with hydrogen peroxide (H_2_O_2_) and analyzed by fluorescence‐activated cell scanning (FACS) at the indicated time points. Statistical differences were determined by an unpaired student's *t*‐test (mean ± SEM, *n* = 2, not significant (ns): *P *>0.05; *P <0.05 and **P <0.01).

To analyze age‐related changes in humans we isolated primary T cells from aged (> 55 years old) and young (20 ‐ 25 years old) individuals. Since accumulation of oxidative damage has been linked to aging we asked whether expression of TXNIP is altered during aging. We observed increased TXNIP expression levels in T cells of aged donors in comparison to T cells isolated from young donors (Fig. 1A,B and Fig. S1A). Analysis of other primary human cells of the hematopoietic system (e.g. hematopoietic progenitor cells [HPC] and monocytes) also showed enhanced TXNIP levels in aged individuals (Fig. 1D,E). Non‐hematopoietic tissues such as liver, showed a marked tendency for upregulation of TXNIP levels in aged individuals (Fig. 1F). Similar effects have also been described in the brain cortices of aged rats 22. Thus, these data suggest a general role for TXNIP in aging. Of note, increased expression of other α‐arrestins during aging was not observed (Fig. S1B,C). Since TXNIP is a negative regulator of TRX‐1 28, 29, 30 we analyzed TRX‐activity. We found that increased TXNIP expression correlated with reduced TRX activity in cells from older individuals (Fig. 1C). This suggests that increased TXNIP expression may increase the pro‐oxidative intracellular status during aging. To further test this idea, Jurkat T cells were stably transfected with an inducible TXNIP shRNA. TXNIP knock‐down (Fig. 2A,B) improved resistance to oxidative stress induced by hydrogen peroxide (H_2_O_2_), further substantiating a role for TXNIP in redox regulation (Fig. 2C).

### Drosophila is an aging model comparable to humans

**Figure 3:**
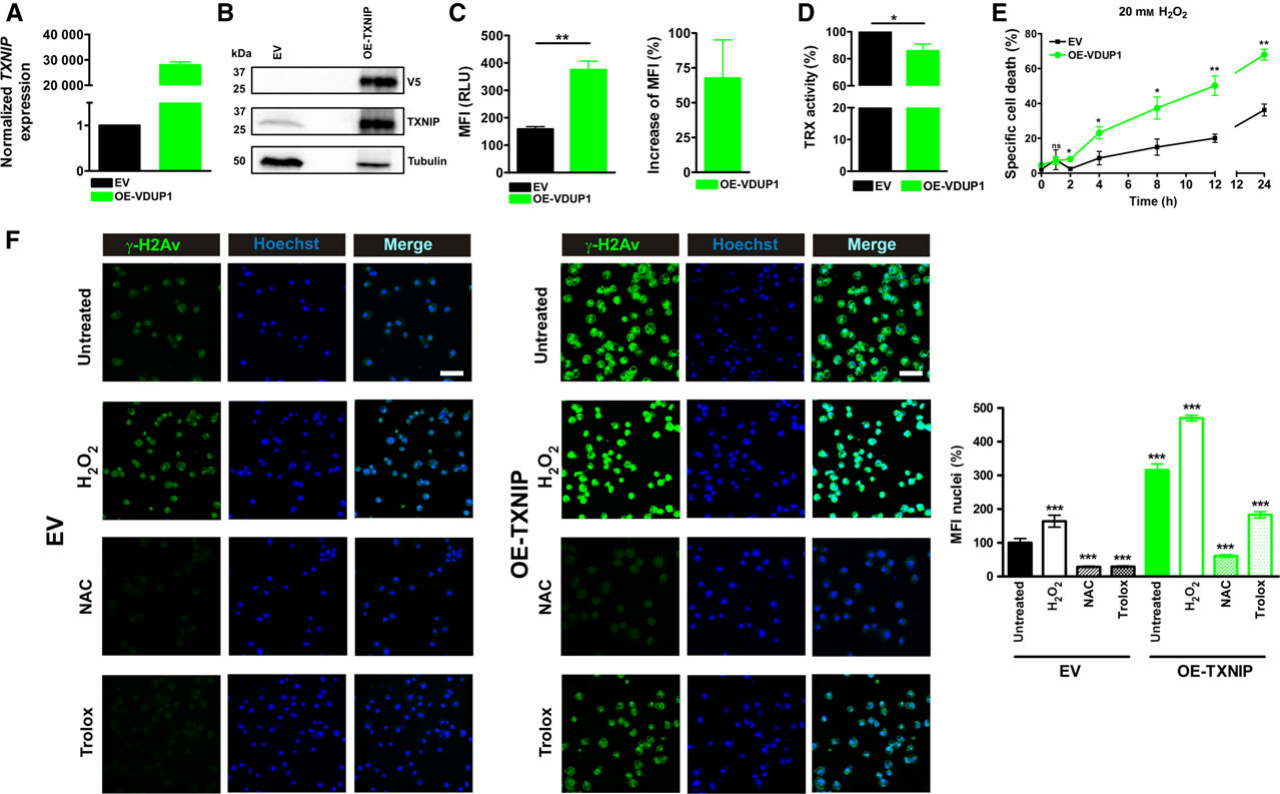
TXNIP overexpression in *Drosophila* Schneider‐2 (S2) cells decreases TRX‐activity, increases ROS generation and susceptibility to oxidative stress and sensitizes cells to oxidative DNA damage. (A,B) TXNIP expression was analyzed by qPCR (A) or by Western blot analysis (B). (B) 80 μg protein lysate of EV (empty vector) and 30 μg of OE‐TXNIP lysate were loaded on the gel and probed with the indicated antibodies. (C) For determination of reactive oxygen species (ROS) generation, cells were stained with H_2_DCF‐DA and an increase in mean fluorescence intensity (MFI) was analyzed by fluorescence‐activated cell scanning (FACS, left panel) and normalized to empty vector controls (EV; right panel). Statistical differences were determined by an unpaired student's *t*‐test (mean ± SEM, *n* = 3, **P < 0.01). (D) Thioredoxin (TRX) activity in lysates was normalized to EV. Statistical differences were determined by Welch's *t*‐test (mean ± SEM, *n* = 3, *P < 0.05). (E) Cell death was induced by treatment with hydrogen peroxide (H_2_O_2)_ and analyzed by FACS. Statistical differences were determined by an unpaired student's *t*‐test (mean ± SEM, *n* = 3, not significant (ns): *P* > 0.05, *P < 0.05 and **P < 0.01). (F) DNA damage was analyzed by immunostaining for phospho‐γH2Av. γH2Av is shown in green and nuclei are shown in blue by Hoechst33342 staining. Scale bar, 10 μm. The bar diagram shows % of MFI of 10 nuclei and was normalized to EV. Statistical differences were determined by an unpaired student's *t*‐test (mean ± SEM, *n* = 10, ***P < 0.001).

**Figure 4:**
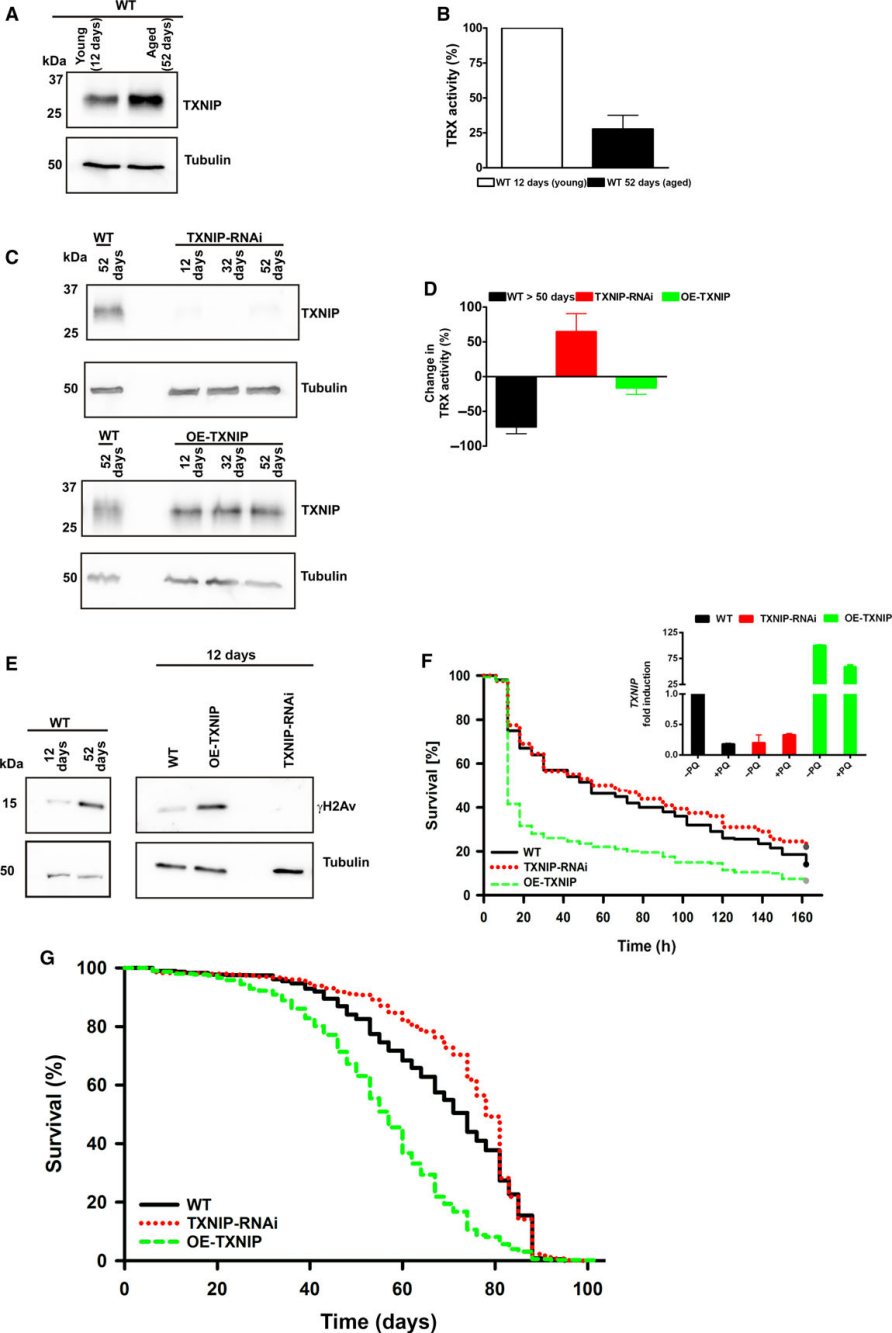
Overexpression of TXNIP in female flies leads to reduced resistance to oxidative stress and to a significantly shortened median lifespan. (A) Endogenous TXNIP expression (WT flies) was analyzed by Western blot analysis at the indicated time points after eclosion. (B) Thioredoxin (TRX) activity of endogenous TXNIP (WT flies) was analyzed in aged flies and normalized to young flies. (C) Transgenic TXNIP expression was analyzed by Western blot analysis at the indicated time points after ecolsion. (D) Thioredoxin (TRX) activity was calculated as change in TRX activity and was normalized to young wildtype (WT) flies (12 days). (E) Phosphorylation of γH2Av (indicating DNA damage) was analyzed by Western blot in young and aged WT flies (left panel) or young OE‐TXNIP and TXNIP‐RNAi flies and compared to WT of the same age (right panel). (F) Enhanced TXNIP expression reduced oxidative stress resistance. Survival curves of female flies exposed to paraquat (PQ, a redox‐cycling agent) are shown (*n* = 200 per genotype, Log‐Rank‐Test: *P* < 0.001). TXNIP expression after paraquat (+PQ) treatment was analyzed by qPCR and normalized to WT flies without (‐PQ) treatment (inset)**.** (G) Enhanced TXNIP expression decreased lifespan of female flies as shown by their survival curves. Knock‐down of TXNIP in females led to an increase in lifespan (*n* = 705 per genotype, Log‐Rank‐Test: *P* < 0.001).

Since *Drosophila melanogaster* express a TXNIP homologue called VDUP1 and are a widely accepted aging model comparable to humans 25, 31, 32, 33 we used them to test whether TXNIP directly affects organismal aging. Importantly, as seen in humans, *Drosophila* TXNIP is upregulated during aging in *Drosophila* (Fig. 4A). To determine whether TXNIP is a pivotal regulator of aging, we investigated the role of *Drosophila* TXNIP *in vitro* and *in vivo*. We generated *Drosophila* Schneider‐2 cells (S2) overexpressing *Drosophila* TXNIP (OE‐TXNIP) (Fig. 3A,B). To elucidate the role of TXNIP in regulating redox equilibrium*,* we determined basal levels of reactive oxygen species (ROS). OE‐TXNIP cells showed an increase of up to 68% in basal ROS levels compared to empty vector (EV) transfected control cells (Fig. 3C). In addition, TXNIP overexpression decreased TRX activity by 14% relative to EV transfected cells (Fig. 3D). Hence, TXNIP overexpression in S2 cells led to a shift to a pro‐oxidative cellular status. Of note, overexpression of TXNIP did not result in changes in expression levels of *Drosophila* TRX homologues or other redox‐related genes (Fig. S2). Impairment of the cellular redox balance has been shown to render cells more susceptible to oxidative stress 1, 34. Therefore, we tested resistance to oxidative challenge by assaying specific cell death after H_2_O_2_ addition. Overexpression of TXNIP sensitized S2 cells towards oxidative stress (Fig. 3E). Increased ROS levels and an attenuated oxidative defense can lead to damage of macromolecules such as proteins, lipids and nucleic acids. Accumulation of DNA damage is known to be involved in aging 5, 35, 36. An indicator for DNA double strand breaks and oxidative DNA damage is phosphorylation of histone H2A. To study the effect of TXNIP expression on the generation of oxidative DNA damage, we investigated γH2Av phosphorylation by immunofluorescence. While unchallenged S2 cells overexpressing TXNIP already showed an increase in γH2Av phosphorylation compared to EV transfected control cells (Fig. 3F, upper panel), H_2_O_2_ treatment further increased DNA damage, especially in cells overexpressing TXNIP (Fig. 3F, middle panel). The ROS‐scavengers *N*‐acetyl cysteine (NAC) and Trolox both blocked γH2Av phosphorylation efficiently (Fig. 3F, lower panel). This clearly indicates that TXNIP‐mediated DNA damage is ROS dependent.

### TXNIP expression regulates lifespan of Drosophila

First, we analyzed endogenous TXNIP levels of young (12 days) and aged (52 days) WT flies (Fig. 4A). This increase in endogenous TXNIP expression also modulates the cellular redox balance, as shown by the reduction in endogenous TRX activity in aged (> 50 days) compared to young (12 days) WT flies (Fig. 4B). To analyze the role of TXNIP in stress resistance and lifespan, we generated TXNIP knock‐down (TXNIP‐RNAi) flies and flies overexpressing TXNIP (OE‐TXNIP) (Fig. 4C). We excluded that TXNIP expression influenced body size and weight in these different genotypes, because these factors can impact lifespan in insects (Fig. S3). Since we had shown that TXNIP expression regulates TRX activity (Figs 1C, 3C and 4B), we investigated whether transgenic TXNIP levels also influence TRX activity *in vivo*. Whole fly lysates were prepared and TRX activity was determined. We observed that enhanced TXNIP (levels OE‐TXNIP) decreases TRX activity whereas low levels of TXNIP (TXNIP‐RNAi) resulted in increased TRX activity in comparison to young wild type (WT) controls (Fig. 4D). Decreased TRX activity led to accumulation of oxidative damage *in vitro* (Fig. 3F). Therefore, we compared young (12 days) and old WT flies (> 52 days) to analyze if lower TRX activity also led to DNA damage *in vivo*. Western blot analysis revealed a strong increase in phosphorylated γH2Av in aged WT flies (Fig. 4E, left panel). Thereafter, DNA damage in young flies (12 days) was analyzed. Already young WT flies showed minor increased γH2Av phosphorylation indicating low but detectable levels of DNA damage. However, young flies overexpressing TXNIP showed a massive increase in γH2Av phosphorylation indicating severe DNA damage. Remarkably, nearly no γH2Av phosphorylation could be detected in young RNAi‐TXNIP flies. Thus, downregulation of TXNIP results in a better protection to DNA damage even in comparison to normal (WT) expression levels of TXNIP (Fig. 4E, right panel). Next, we investigated the role of TXNIP expression in resistance to oxidative stress *in vivo*. We fed flies with the redox‐cycling agent paraquat (PQ) and monitored survival under these oxidative stress conditions. OE‐TXNIP flies showed significantly reduced survival upon PQ treatment (Fig. 4F). Interestingly, knock‐down of TXNIP had only mild effects on oxidative stress resistance compared to WT (Fig. 4F). This is explained by downregulation of endogenous TXNIP expression upon exposure to oxidative stress (Fig. 4F, inset). Of note, survival under starvation conditions in flies (fed with water only) was not altered in OE‐TXNIP or TXNIP‐RNAi flies (Fig. S3B).

Endogenous TXNIP is upregulated during aging in flies (Fig. 4A). Increased TXNIP expression led to downregulation of TRX activity (Fig. 4B,D), increased DNA damage (Fig. 4E) and impaired resistance towards oxidative stress (Fig. 4F). All these changes are important events that occur during aging 1, 5, 34. Thus, TXNIP is a promising candidate to regulate lifespan in flies. To investigate whether TXNIP expression is directly involved in aging we analyzed the lifespan of the different fly strains. We found that overexpression of TXNIP significantly shortened median lifespan (Fig. 4G, Log‐Rank‐Test: *P* < 0.001), whereas depletion of TXNIP significantly extended median lifespan (Fig. 4G, Log‐Rank‐Test: *P* < 0.001). Lifespan shortening and the stress‐resistance phenotypes noted above were observed in both sexes, but significant lifespan extension was observed only in females, the standard subject for *Drosophila* longevity assays 37, 38. Thus, the increased expression of TXNIP during aging correlates with a regulatory effect in which TXNIP levels negatively affect *Drosophila melanogaster* lifespan.

## Discussion

In summary, we identified that the redox regulator TXNIP controls lifespan and resistance towards oxidative stress. We propose a novel mechanism in which TXNIP decreases TRX activity and, thereby, impairs cellular redox homeostasis during the aging process. Data from both humans and *Drosophila melanogaster* support this assumption. In agreement with the “free radical theory of aging” 1, our data from *Drosophila* show for the first time that the TXNIP‐induced shift to a pro‐oxidative cellular environment facilitates DNA damage (Figs 3, 4). In accordance with the idea that oxidative stress and DNA damage are major factors influencing deterioration of cellular functions during aging 5, 35, 36, 39, lowered expression of TXNIP improved cellular resistance to oxidative stress (Fig. 3) and extended lifespan (Fig. 4G). Our finding that TXNIP expression increases during aging is all the more interesting when one considers that increased expression of TXNIP is also observed in age‐related diseases such as diabetes 40, 41, 42 and neuronal degeneration 43, 44. Aging of the population has major implications for health care resources and workforce productivity 45. Therefore, improvements in healthy and productive aging are of major interest. Manipulation of TXNIP expression/activity could be a promising option to promote healthy aging.

## Author contributions

TO designed and performed experiments, analyzed the data, discussed results and wrote the paper. JB, SZ, AS, DR, LK, WW designed and performed experiments and analyzed data. BAE contributed to experimental design, analyzed data and edited the manuscript. KG and PHK initiated, designed, guided the research and edited the manuscript.

## Supporting information


**Fig. S1.** Expression of arrestin family members is not changed upon aging.
**Fig. S2.** Gene expression of *Drosophila* redox‐related genes is not changed upon TXNIP overexpression.
**Fig. S3.** TXNIP expression levels in female flies do not influence body size or weight and do not influence lifespan under starvation of the generated fly stains.
**Table S1.** Summary of qPCR primer sequences.
