# Supplementary material for: Enhanced expression of thioredoxin‐interacting‐protein regulates oxidative DNA damage and aging
Source: FEBS Lett. 2018 Jun 27;592(13):2297–307. doi: 10.1002/1873-3468.13156 (PMC6099297; doi:10.1002/1873-3468.13156)
Supplement: Supplementary file 1 — Fig. S1. Expression of arrestin family members is not changed upon aging. Fig. S2. Gene expression of Drosophila redox‐related genes is not changed upon TXNIP overexpression. Fig. S3. TXNIP expression levels in female flies do not influence body size or weight and do not influence lifespan under starvation of the generated fly stains. Table S1. Summary of qPCR primer sequences. [file FEB2-592-2297-s001.pdf]

Supporting Information

Supplement Figures Legends

Supplementary Fig. S1. Expression of arrestin family members is not changed upon aging.

(A) The diagram shows the quantification of three different Western blots. TXNIP signal was divided by the values of the respective actin signal and normalized to young donors. (B,C) α-Arrestin family members ARRB1 (B) and ARRB2 (C) show similar mRNA expression in T cells of young and aged blood donors as determined by qPCR. Statistical significance was determined by an unpaired student's t-test (mean ± s.e.m., not significant (ns): $p>0.05$).

Supplementary Fig. S2.

Gene expression of *Drosophila* redox-related genes is not changed upon TXNIP overexpression.

(A-G) Gene expression of the indicated genes was determined by qPCR. Results are displayed as relative gene expression compared to empty vector (EV) control transfected cells and were normalized to *rp49* expression. *Trx-2A* = Thioredoxin 2A, *Trx2B* = Thioredoxin-2B, *TrxT* = Thioredoxin-T, *dhd* = deadhead, *Cat* = Catalase, *SOD1* = Superoxide Dismutase 1 and *SOD2* = Superoxide Dismutase 2.

Supplementary Fig. S3. TXNIP expression levels in female flies do not influence body size or weight and do not influence lifespan under starvation of the generated fly stains.

(A) Representative image of flies showing equal body size (upper panel). Flies were anesthetized and weight was determined (lower panel). Statistical significance was determined by an unpaired student's t-test (mean ± s.e.m., n=3, not significant (ns): $p>0.05$). (B) Survival curves of flies under starvation conditions (n=150 per genotype). Of note, flies were kept without any food, only water was supplied.

Supplement Figures

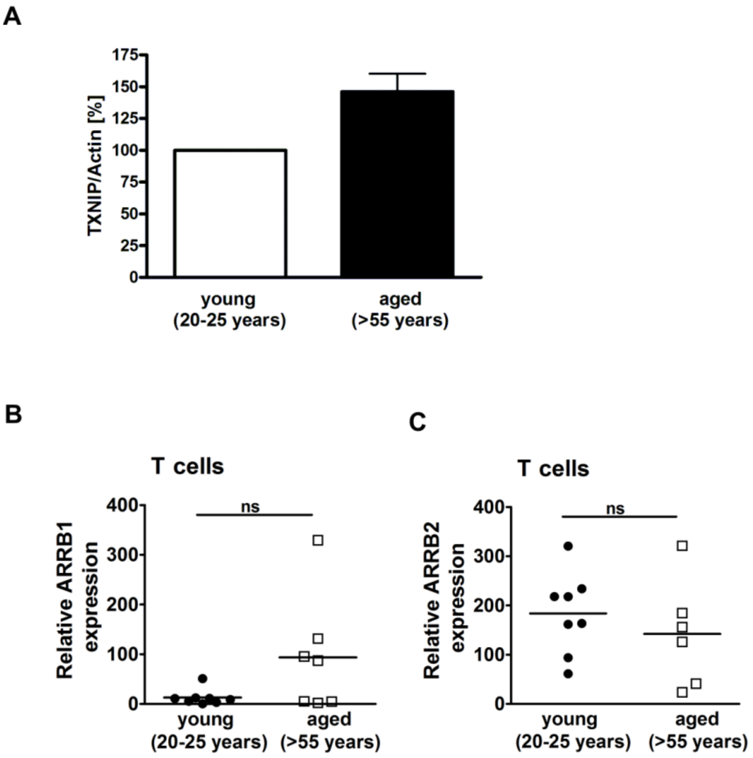

Supplementary Fig. S1

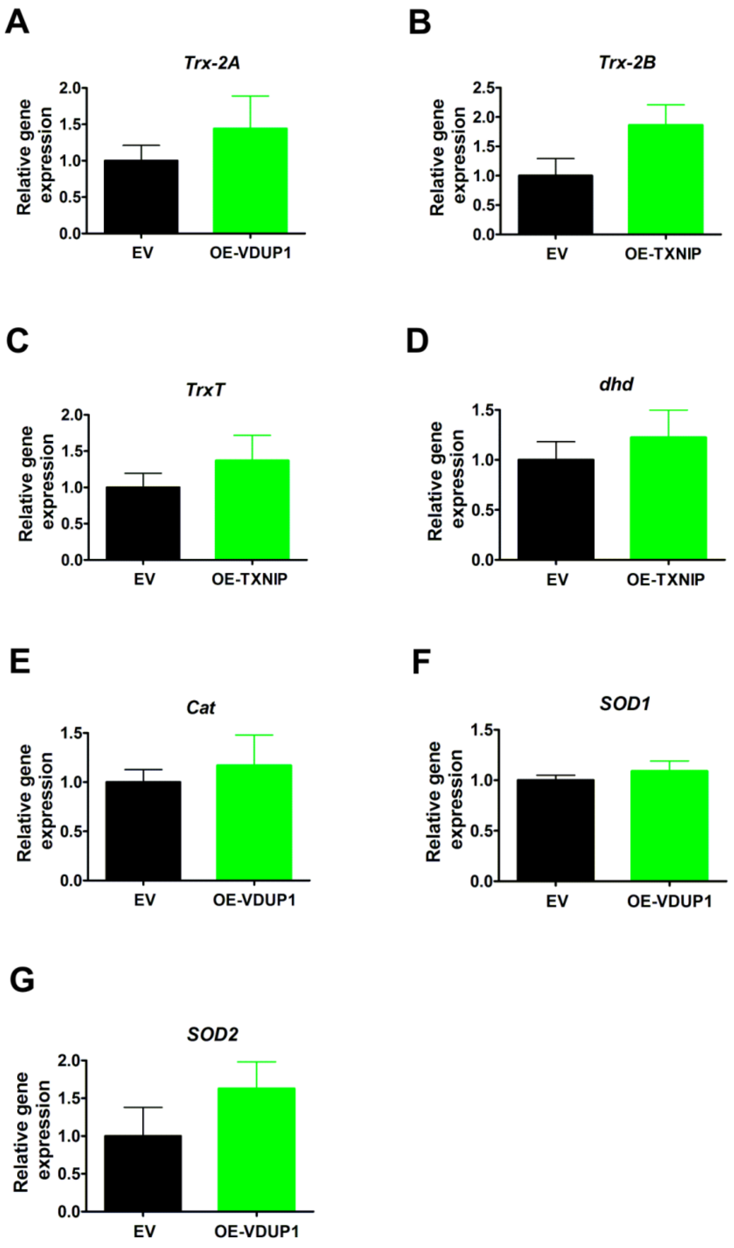

Supplementary Fig. S2

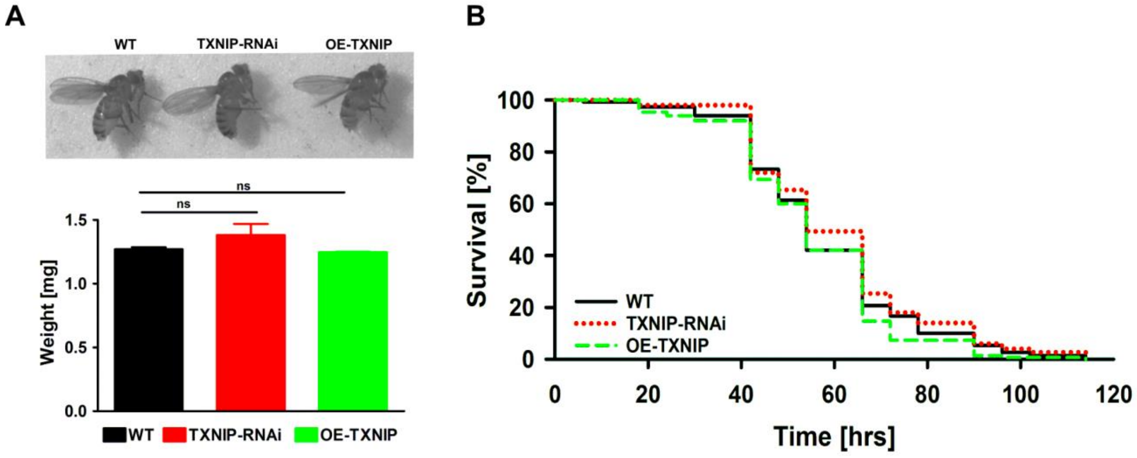

Supplementary Fig. S3

Table 1

Summary of qPCR primer sequences.

Primer name	Sequence 5’-3’
GAPDH hu forw.	GCA AAT TCC ATG GCA CCG T
GAPDH hu rev.	TCG CCC CAC TTG ATT TTG G
hTXNIP forw.	TCA TGG TGA TGT TCA AGA AGA TC
hTXNIP rev.	ACT TCA CAC CTC CAC TAT C-
ARRB1 hu forw.	AGT GGC CGT GGA ACT GCC CTT CA
ARRB1 hu rev.	GGA ACT TCC CGA TGC GGG GGT TC
ARRB2 hu forw.	GGG CAA GCG GGA CTT CGT AGA
ARRB2 hu rev.	TGC GGT CCT TCA GGT AGT CAG GG
OE-TXNIP dros forw.	AAG GCA TCC CTC ACT GAG AC
OE-TXNIP dros. rev.	AGC TCC CGT TTT TCC GTT TG
Trx-2A dros forw.	GCG ACG TGC TCA TTT TGG TAA
Trx-2A dros rev.	GGA TGG GAG ATG TGG AGA CG
Trx-2B dros forw.	GCA TAG TGG CCA GCA AAA CC
Trx-2B dros rev.	ACA GGC TCA CGC TTC TCA TT
TrxT dros forw.	TGT ACC CAG TGC GGA ACA AG
TrxT dros rev.	TCG TCC ACG TTC ACC TTG AG
Dhd dros forw.	GTA AGC GCG AGA TGT GGG TA
Dhd dros rev.	CGC GCC ACA AAT CAT AGC AT
SOD2 dros forw.	*AGCGAAATAACGAGAACGTAAGC*
SOD2 dros rev.	CCGCCAGGCTTGCAGTTTG
SOD1 dros forw.	ACACGAGCTGAGCAAGTCAA
SOD1 dros rev.	CAGTGGCCGACATCGGAATA
rp49 forw.	CGG ATC GAT ATG CTA AGC TGT
rp49 rev.	CGA CGC ACT CTG TTG TCG
